# A review of reviews exploring patient and public involvement in population health research and development of tools containing best practice guidance

**DOI:** 10.1186/s12889-023-15937-9

**Published:** 2023-06-30

**Authors:** Soo Vinnicombe, Mayara S. Bianchim, Jane Noyes

**Affiliations:** School of Medical and Health Sciences, Bangor University, Bangor, UK

**Keywords:** Public and patient involvement, Involvement, PPI, Population health research, Review of reviews, Guidance

## Abstract

**Introduction:**

Patient and public involvement (PPI) is increasingly seen as something that is integral to research and of importance to research funders. There is general recognition that PPI is the right thing to do for both moral and practical reasons. The aim of this review of reviews is to examine how PPI can be done ‘properly’ by looking at the evidence that exists from published reviews and assessing it against the UK Standards for Public Involvement in Research, as well as examining the specific features of population health research that can make PPI more challenging.

**Methods:**

A review of reviews and development of best practice guidance was carried out following the 5-stage Framework Synthesis method.

**Results:**

In total 31 reviews were included. There is a lack of current research or clarity around Governance and Impact when findings are mapped against UK Standards for Public Involvement in Research. It was also clear that there is little knowledge around PPI with under-represented groups. There are gaps in knowledge about how to ensure key specific attributes of population health research are addressed for PPI team members – particularly around how to deal with complexity and the data-driven nature of the research. Four tools were produced for researchers and PPI members to further improve their PPI activity within population health research and health research more generally, including a framework of recommended actions to address PPI in population health research, and guidance on integrating PPI based on the UK Standards for Public Involvement in Research.

**Conclusions:**

Facilitating PPI in population health research is challenging due to the nature of this type of research and there is far less evidence on how to do PPI well in this context. The tools can help researchers identify key aspects of PPI that can be integrated when designing PPI within projects. Findings also highlight specific areas where more research or discussion is needed.

**Supplementary Information:**

The online version contains supplementary material available at 10.1186/s12889-023-15937-9.

## Background

The focus of this review of reviews is on Public and Patient involvement (PPI) in population health research and the subsequent development of best practice guidance to further improve PPI practice. PPI is increasingly seen as something that is integral to research and of importance to research funders. For our purposes, PPI is defined as ‘research that is developed with the public’. Specifically, patients or members of the public with relevant lived experience can be involved at any stage of the research project, including the research design, delivery and dissemination. When done well, PPI is fundamental to protect and promote the interests of patients and the public, and it also helps to create research that is more relevant with clearer outcomes and impact [1]. More practical benefits of including PPI in research are reduced waste and improved quality [2]. High quality impactful research addressing population health issues with planned and integrated PPI is needed now more than ever given the recent global Covid 19 pandemic where research was commonly conducted in isolation of PPI, and the public lacked trust in some of the evidence produced (such as compulsory stay at home orders and mask wearing) [3].

Defining when PPI is done well and has been effective has also been challenging for researchers to articulate. Research funders commonly set out expectations for including PPI in research studies but there is less acknowledgement as to what is sufficient PPI, what ‘good’ PPI looks like and what impact PPI has had on the research outcomes. In the UK, six standards [4] have been published as to what ‘good PPI’ looks like in relation to quality and consistency of involvement (see methods section for further details). Researchers are also increasingly reporting the outcomes of PPI on their research as well as the outcomes of the study [5, 6].

It is important to highlight that public involvement is not the same as taking part in a study as a research participant. Public involvement is not the same as public engagement. The latter refers to the process of engagement to obtain feedback and sharing research findings with the public [1]. There is however sometimes confusion between what constitutes public engagement compared with involvement. In some countries, such as Canada, it is also common to use ‘public engagement’ to refer to public involvement [7]. Similarly, the lines between stakeholder representation and public or patient representation can sometimes be blurred.

### Population health research

‘Population health’ is associated with several definitions and nuances and there is overlap with public health and aspects of more general health research. The King’s Fund defined population health as: Research that is designed with the aim to benefit the health of a population. It focuses on improving outcomes such as physical and mental health and wellbeing of a determined population while reducing health inequalities. It can include the goals of reducing illnesses or/and delivering health and care services. Population health focuses on the wider determinants of health and it can involve communities and partner agencies [8].

‘Public health’, by comparison, can be defined as: Activities to strengthen public health capacities and service aims to provide conditions under which people can maintain their health, improve their health and wellbeing, or prevent the deterioration of their health. Public health focuses on the entire spectrum of health and wellbeing, not only the eradication of particular diseases [9].

Some refer to Public Health (note the capitalisation) as specifically about activities and interventions carried out by government agencies, health professionals, or other centralised bodies whereas population health includes other, non-health related, influences such as housing, transport and education. In reality, these various definitions can oversimplify our understanding and a rigid adherence to a perceived difference between the terms may serve to disguise relevant information about successful PPI activity. For Diez-Roux, what really matters are the answers and actions arising from the questions raised regarding the health of the public, and everything else is a semantic discussion [10].

### Specific challenges of integrating PPI in population health research

Population health research, or health research that considers population level questions, provides challenges in terms of PPI that are not always present in condition-specific research projects. For example:

- Duration. Population health research often looks at health variables across a long period of time. This makes recruiting and retaining suitable PPI representation across the length of the project more challenging. Changes in personnel, in all parts of the research team and partners, can be expected in any project.
- Complexity. Population health is often multi-disciplinary and looks at health as the product of multiple determinants (such as biology, genetics, behaviours, social and environmental aspects) as well as looking at their interactions among individuals and groups and across time and generations. With all these different variants involved it can be difficult for a lay person to understand the complexity – or, to put it another way, for the researchers to explain the research in a way that a lay person can understand. It may often be the case that a different skill set, and therefore potentially a different person, is necessary at different stages of the research or for different workstreams – something that applies to researchers as well as to PPI representatives.
- Data-driven. Population health projects are often driven by large datasets and can involve knowledge of algorithms, advanced statistics, and analytical techniques that can be unfriendly to the non-mathematically minded. It can be a challenge for researchers to ‘translate’ both the process and the outcomes of their research in terms that can be more widely understood. This is one reason why PPI can be so helpful in such projects. For example, helping to design dissemination activity that is meaningful to a broad audience.
- Representation. Population health research often addresses large and diverse population groups within the populations being researched, which raises issues about the PPI being representative. Even within disease-specific studies it is often difficult, if not practically impossible, to recruit someone who truly represents the breadth of people with a certain condition. Once that issue is expanded out to wider populations, the issue of true representation is multiplied many times. Representation becomes particularly difficult with certain demographic groups which may be grouped together for convenience, but which might hide a variety of differences. A prime example of this is the involvement of ethnic minority communities – recruiting a single person of ethnic minority background risks subsuming important differences according to specific cultural, genetic, class, education and other factors. There is also an ongoing debate about terminology such as ‘hard to reach’, ‘under-represented’, ‘seldom heard’ and ‘under-served’ which often have problematic resonances (11). The definition of ‘under-served’ is highly context-specific; it will depend on the population, the condition under study, the question being asked by research teams, and the intervention being tested. No single, simple definition can encompass all under-served groups (12).

### The need for a review of reviews and new guidance

As described above, population health presents specific challenges for researchers and there is a lack of guidance on doing PPI well in population health research. Scoping searches identified a number of reviews of PPI involvement covering population health, public health as well as other more general reviews that included population and public health studies of interest. None of the published reviews had a specific focus on what worked to deliver optimal PPI in population health research. As core researchers with the National Centre for Population Health and Wellbeing Research in Wales (NCPHWR) (https://ncphwr.org.uk/), we were tasked with developing guidance to fill this identified gap. We therefore decided to undertake a review of reviews to explore the challenges and solutions to carrying out PPI well in population health research and to produce guidance to support further development of PPI practice in this field. Four tools reporting best practice guidance and highlighting key resources were subsequently developed to further improve the quality of PPI activities in population health research.

## Materials and methods

This review of reviews assembled and interpreted the evidence on PPI involvement in population health research. Question formulation was underpinned by the ECLIPSE (**E**xpectation, **C**lient Group, **L**ocation, **P**rofessionals and **Se**rvice) framework that is acknowledged to be most suitable for searching for health policy or health management information [13].

We developed the following question: What evidence exists concerning the successful development, implementation and evaluation of patient and public involvement activity or models in population health research in the UK and equivalent health systems?

### Inclusion criteria

- Type of study: systematic and other reviews that focus on the concept of, or approaches to, PPI and/or PPE (patient and public engagement) across population health, public health, health and social care. Limited to systematic reviews, narrative reviews, literature reviews, bibliometric reviews, scoping reviews and meta-analyses. Quantitative, qualitative and mixed-methods reviews were of interest.
- Setting: any organisational setting that includes population health, public health, health or social care aspects (e.g., primary care, mental health, hospital, tertiary care, voluntary, etc.).
- Type of involvement: not just being part of the research as a participant but being involved in part or all of the following stages – research development, research monitoring, research analysis and dissemination.

### Exclusion criteria

- Articles not in English.
- Reviews published before 2010. However, the timeframes for the primary studies included in the reviews varied and could go back to the inception of various databases. This timeframe was considered appropriate as public and patient involvement is something that has been developing rapidly in recent years and was not really established as a well-recognised term before then.

### Search Strategy

An information scientist undertook the initial search of the Medline and PubMed databases. The full search strategy is included in supplementary file 1. The Involve Evidence Library was searched for ‘systematic reviews’. Note that this library only includes references up to 2015. The original search was done in May 2020 with a follow up search (stages 2 and 3) carried out early in September 2021 to pick up new reviews up to end of August 2021.

### Screening

**Fig. 1:**
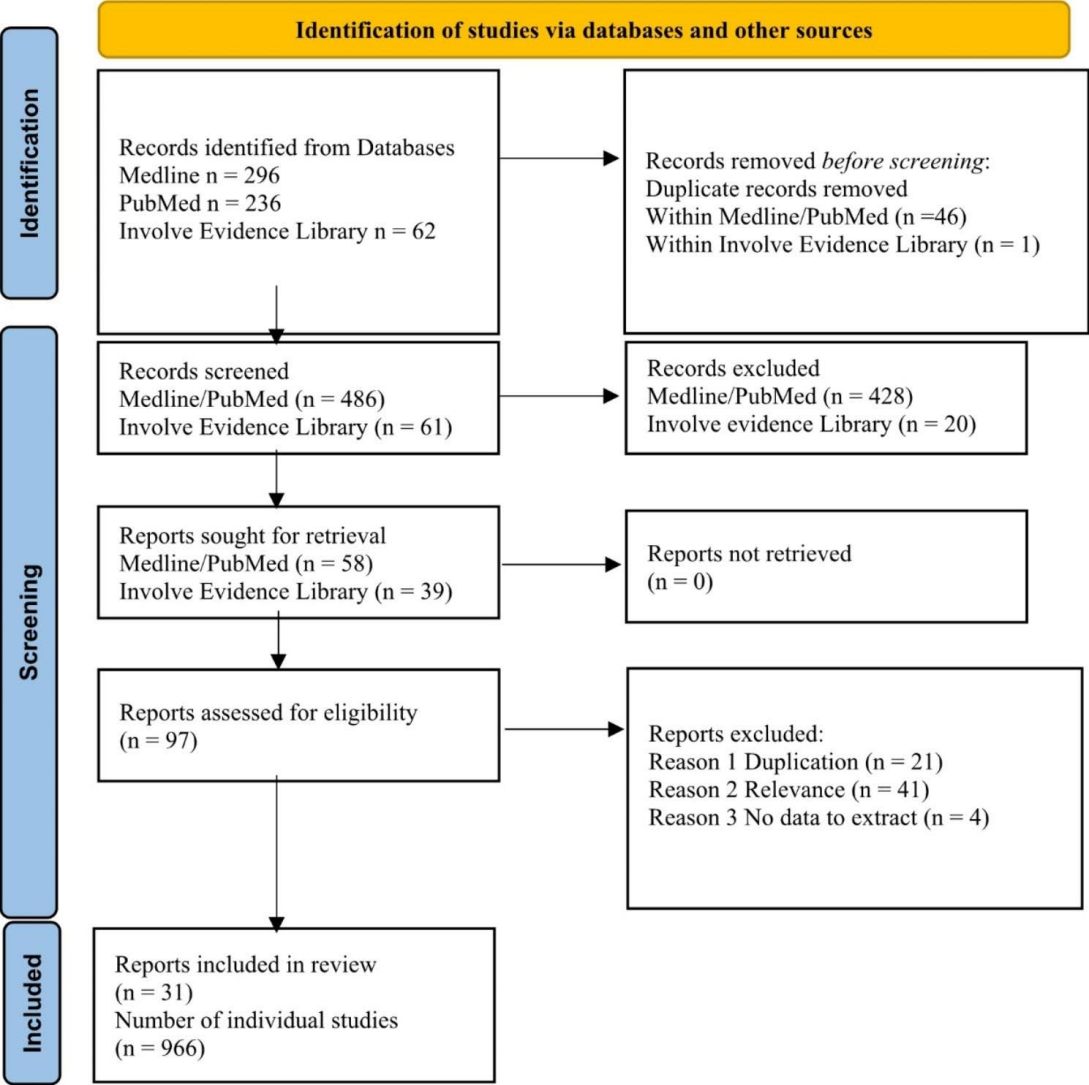
PRISMA Flow Diagram. * This number is incomplete due to missing information on some papers. Duplicates have been removed (original n = 1222)

Titles and abstracts were screened to identify reviews that met the inclusion criteria. Potentially relevant reviews were retrieved and the full text assessed for inclusion (Fig. 1). The process was undertaken by SV and independently checked by JN.

### Quality appraisal

Originally the AMSTAR2 [14], method was trialled on six reviews but as most of the included reviews were qualitative rather than quantitative many of the AMSTAR2 domains did not apply so we switched to using CASP for systematic reviews [15]. Included reviews were quality appraised by SV and independently checked by JN (see supplementary file 2 for results of quality assessments). Reviews were not excluded at this stage on methodological grounds as the focus was on PPI processes reported in the review.

### Data extraction and synthesis

Studies included in source reviews were mapped for duplication and this was taken account of in the analysis and synthesis. As this review of reviews did not require a transformative method of data synthesis to better understand the descriptive accounts of PPI in the source reviews, we selected the aggregative 5-stage Framework synthesis method for integrating evidence of interest from diverse review designs and to identify examples of best practice.

It is a matrix-based method involving the construction of a priori thematic categories into which data can be coded [16]. The five stages are:

- Familiarisation.
- Identifying a thematic framework.
- Indexing.
- Charting.
- Mapping and interpretation.

Initial data extraction was carried out against a framework designed by the authors based on close examination of background literature, initial review readings and a desire to identify best practice. (Table 1).

**Table 1:** Initial framework: headings and details

Main info	Title
	Authors
Extracted information	Year published
	Type of review
	Area of focus
	No. of studies
	No. of papers
	Full list
	Databases searched
	Other searches
	Years searched
	Exclusions
	Geography
	Methods used
	Included PPI in own review
Why do PPI?	Attribute
	Who benefits?
	Evidence for
	Evidence against
How to do PPI – especially in	Attribute – barrier
population health research	Stage affected
	Mitigation
	Attribute - facilitator
	Stage affected
	Good practice
Terminology	Types of PPI
	Stages of research
	Other
Other	Gaps in Knowledge
	Country specific legislation/ guidance
	Case studies?

Extracted data were subsequently mapped against a second framework (Table 2) and matched against the UK Standards for Public Involvement to identify examples of solutions to problems and best practice [4].

**Table 2:** Secondary framework: thematic mapping

Challenges			Solutions		
Study id	Problem	Consequence	Study id	Solution	Details

The UK Standards for Public Involvement are:

- Inclusive Opportunities - Offer public involvement opportunities that are accessible and that reach people and groups according to research needs.
- Working Together - Work together in a way that values all contributions, and that builds and sustains mutually respectful and productive relationships.
- Support and Learning - Offer and promote support and learning that builds confidence and skills for public involvement in research.
- Governance - Involve the public in research management, regulation, leadership and decision making.
- Communications - Use plain language for well-timed and relevant communications, as part of involvement plans and activities.
- Impact - Seek improvement by identifying and sharing the difference that public involvement makes to research [4].

### Development of tools containing best practice guidance

Selected tables developed to display examples of best practice mapped against the UK standards for PPI as part of the mapping and charting of the Framework synthesis easily translated with minor editing into tools outlining best practice principles for researchers and PPI (Supplemental file 4). These resources were shared with members of the NCPHWR and PPI members for feedback.

### Public and patient involvement

This review of reviews included PPI input, specifically, the draft review was read and commented on several times throughout its development by two PPI members from the Centre for Population Health Patient and Public Involvement Advisory Group. This PPI group meets quarterly to help set the strategic direction for PPI within the Centre.

## Results

Thirty-one reviews were included covering around one thousand individual studies, which were mainly based in the UK or USA. We took note of any duplication of studies across reviews to ensure that we were not double counting the evidence.

The studies covered a range of settings and subject areas (see supplementary file 3 for a description of all included studies). Reviews varied in quality (see supplementary file 2 for results of quality assessments) but as the review methods and findings were not the primary phenomenon of interest, we did not place a lot of emphasis on the quality of the source reviews when interpreting findings.

Specifically, the reviews covered, to varying degrees, three out of the four challenges, outlined earlier, that set population health research apart from many other research types.

**Representation** was extensively discussed in the studies reviewed. It is an aspect of PPI that does not have a simple solution for any type of research project. For population health projects that tend to be longer in duration, it may be that different people need to take part in different periods of the project and, for complex projects, that different people need to be involved in different work streams. Boote [17] noted a concern that PPI representatives taking part in research over time may become ‘professionalised’ and come to see things from the point of view of the research team rather than as a member of the public or patient demographic.

**Complexity** was also discussed when talking about support and learning requirements for PPI members. Population health projects are often highly complex but, given the right support and training, that is not a sufficient reason to exclude PPI activity.

The **data-driven** aspect was touched upon mainly in terms of ensuring that project specific training and support was available. Many population health projects include aspects of Big Data which can add a layer of difficulty to PPI activity, but which can also be addressed by considering tailored training and support. Having non-data experts involved in such projects may help when designing dissemination and communication activities around the project so that they can eventually be more accessible to a wider audience.

**Duration** was the only aspect that was not specifically discussed in the reviews and in finding solutions. It is possible to postulate that building relationships and strong ways of working together may help to address this issue. But also, that acknowledging upfront the changing requirements of a long-term project will help researchers to plan accordingly – including planning for long term PPI.

### Common issues across PPI activity in population and other types of health research

There are several aspects of PPI activity that are common across various types of health research, including, but not exclusive to, population health research.

### Challenges

Just over half of the reviews (18 out of 31 [18–35]) noted a range of potential challenges with PPI that were reported to stand in the way of the successful development, implementation and evaluation of patient and public involvement activity or models in health research in the UK and equivalent health systems.

Consolidation of the challenges reported in the reviews suggested that the following (Table 3) were the key issues. These have been grouped into appropriate headings.

**Table 3:** Full list of challenges identified

Heading	Sub-heading	Reviews
**Resources**	Lack of budget	18, 20, 22–24, 29, 31, 32
	Lack of time	18, 20, 22–24, 29, 31
	Emotional burden on PPI members	18, 24, 25, 29
	Complicated logistics/ infrastructure	20, 23
	Workload too high (on all sides)	24, 27
	Lack of incentives	20
	Lack of preparation	18
	Lack of staff continuity	19
	Lack of support for PPI members	28
	Scope creep^1^	30
**Conflict and control**	Allowing power to be shared with PPI	18–21, 23, 25, 26, 29
	Expectations (from all sides)	18, 20, 24, 25, 31, 33
	Conflicting perspectives	19, 20, 23, 27, 28
	A culture of researchers vs. PPI members	18, 20, 24
	Ethical concerns	28, 29
	Challenging the establishment	18
	Differences within communities	18
	Accepting the legitimacy of PPI	23
	Prioritising personal experience	22
	Scepticism (from all sides)	18
	Unresolved conflict	35
**Knowledge**	Processes	18, 20, 23, 29, 31
	Language/ jargon	18, 19, 22, 23, 31
	Lack of skills or training	18, 23, 27, 28, 29
	Administration issues	21
	Working practices	18
**Representation**	Reflecting the diversity of affected populations	17, 21–23, 27, 29, 31, 34
	Tokenism of PPI (aka box-ticking)	26, 28, 30
	Getting early-stage involvement	21, 26
	Involving children	23
	Protecting anonymity	29
	Accessibility (venues)	32
**Communication**	Lack of meaningful and timely communication leading to disenfranchisement	18, 21, 25
	Difficulty reporting impact of PPI	19, 28, 29
	Building relationships to sustain involvement	20, 23
	Transparency of research process	27
	Building trust (on all sides)	20
	Different values within team	31

Many of these challenges will be even more apparent in population health research where projects tend to face the four challenges of: longer duration, involving more complex and varied processes, alongside issues of big data, and finding appropriate representation to cover the project breadth and length.

### Solutions

Nearly three quarters of the studies (23 out of 31) [7, 20–22, 24–42] noted a range of potential solutions for ensuring that PPI was more likely to be successful.

These proposed solutions have been collated, consolidated and sorted according to the UK Standards for Involvement in Research as follows:

### Inclusive Opportunities

Solution: Offer public involvement opportunities that are accessible and that reach people and groups according to research needs. Research also needs to be informed by a diversity of public experience and insight, so that it leads to treatments and services which reflect these needs.

Eleven reviews mentioned inclusion (21–[22, 24]–[25, 28, 34, 37]–38, 40–42). Key themes are outlined in Table 4 below and explicitly address the problem area of Representation.

**Table 4:** Solutions – Inclusive opportunities

Attribute	Study/Studies	Examples of reasoning
Representation and/or diversity	24, 28, 37, 40–42	Use variety of methods (41) and partners (28) to recruit a range of participants, understand different motivations (24) and gain insight into the community (37), view differing perspectives as valuable (40), recognise and address issues concerning diversity (40), avoid tokenism (24)
Community consultation	22, 28, 34, 37–38, 41	To fit better with wider community context (37), include relevant stakeholders and agencies (37) also clinicians, charities, specialist support services (41) plus patient and advocacy groups (28), be proactive and go out and get involved, don’t expect people to come to you (38), build more meaningful relationships with target population (34)
Accessibility	24–25, 38, 41	Venues should be located for the ease of the participants (24), accessible and meetings should be timed appropriately (41) and include communication aids, breaks and refreshments as appropriate (25) for individual and collective needs (38)
Methods of engagement	21, 25, 41	Online could assist people to be included e.g. illness, time, caring (21), especially working with disabled children and young people be flexible for different abilities and ages and offer choice (25), use variety of methods (41)
Recruit well	24, 41–42	Fit skills and experiences to the project as well (24), recruit through a variety of ways (41), need to be not just representative but also collaborative (42)
Safe environment	25	Consider whether a trusted adult or facilitator is useful (25)

### Working together

Solution: Work together in a way that values all contributions, and that builds and sustains mutually respectful and productive relationships. Public involvement in research is better when people work together towards a common purpose, and different perspectives are respected.

Twenty-one reviews (7, 20–22, 24–25, 27–33, 35–43) discussed aspects of this standard. The main areas of discussion are outlined in Table 5 below and explicitly address the problem area of Conflict and Control.

**Table 5:** Solutions – Working Together

Attribute	Study/Studies	Examples of reasoning
Relationships	7, 20, 22, 24–25, 28–33, 35–40, 42	Manage conflict (32, 37, 42), Take time to build partnerships built on joint ownership, trust, respect and transparency (7, 20, 20, 25, 28–31, 33, 35–40, 42), Empower PPI members by sharing power and knowledge (25, 36, 38–40), Explore risks together (28), Consider capacity of PPI members (28–29)
Resources	7, 22, 24–25, 28–32, 36, 38, 41	Budget/ funding (22, 24–25, 29, 31–32, 36, 38), Time to build relationships, communicate etc. (7, 22, 24–25, 29–31, 36, 38), Use existing PPI resources where available (41), Plan into proposals (28–29), Tailor to project (38)
Engagement	7, 20–22, 24, 27–28, 33, 42	Early on (7, 21–22, 27, 42), Multiple and varied opportunities (19, 33, 42), Appropriate (24, 28), Acknowledge contributions (21, 28, 42)
Clarity	7, 20, 22, 29–30, 33, 40, 42	Roles (7, 20, 22, 29, 40, 42), Expectations (20, 30, 33, 40), Structures (7)
Flexibility	31, 24–25, 28–29, 43	Confidence, personal circumstances and capacity may change over time (21, 25, 29), Keep tasks flexible and include time for training and questions (28, 43), In attitude and approaches to the project (29)

### Support and learning

Solution: Offer and promote support and learning that builds confidence and skills for public involvement in research. Seek to remove practical and social barriers that stop members of the public and research professionals from making the most of public involvement in research.

Seventeen reviews mentioned various aspects of support and learning [7, 20, 22, 25–26, 28–29, 31–33, 36–42]. The findings are shown in Table 6 below, which is split into two sections to reflect differences between support and learning methods, and explicitly addresses the problem area of Knowledge.

**Table 6:** Solutions – Support and learning

SUPPORT - Attribute	Study/Studies	Examples of reasoning
Emotional support	7, 22, 28, 33, 37–38, 41–42	Recognise that experiences may be upsetting (22), Provide safe spaces (37), Provide consistent feedback and support (28), Consider how to deal with anxiety (33)
Practical support	28, 38–40	Think about details e.g. childcare, food, location, transport, compensation, timings (39), Have strategies for when people are ill/ can’t take part (28)
Structural support	20, 29, 40	Make sure key project individuals support PPI (20), Provide structures that support PPI (40), Include relevant institutions such as charities, volunteer groups etc. (29)
Specific support	33, 37	Ensure support specific to topic area (33) and to their individual involvement (37).
**LEARNING - Attribute**	**Study/Studies**	**Examples of reasoning**
As appropriate	7, 22, 31, 36–37, 40–42	Make learning relevant to the specific context of the research (7, 30, 37) and at the appropriate level for the PPI member (37) to allow full participation (42) and to build participant capacity (22)
Formal knowledge	20, 29, 36, 38	Formal development of knowledge and skills (20), supporting participants to be informed and make informed decisions (29) and to understand specific parts of the research process and/or context (36)
Research methods	26, 36, 41–42	Training in research components to give confidence in their involvement (36) and to explain ‘rules’ and constraints of research (26)
Variety of learning methods	28, 33, 38–39	Use a variety of methods such as supervision, mentoring, formal, workshops and team based (39), include everyone on the team if possible (28, 38)
Share knowledge	36–37	Acknowledge that knowledge and experience flow both ways and make ways to facilitate that flow (37)
General	25, 29, 32, 38	Provide, support and fund training and learning opportunities (29).

### Governance

Solution: Involve the public in research management, regulation, leadership and decision making.

Public involvement in research governance can help research be more transparent and gain public trust. This section explicitly addresses the problem area of Conflict and Control. Only three of the reviews mentioned governance [7, 28, 39]. They discuss the need for shared decision-making (at every level), power and leadership, in order to lead to a culture of deeper involvement. As limited suggestions were reported there is no table for this section.

### Communications

Solution: Use plain language for well-timed and relevant communications, as part of involvement plans and activities. Communicate with a wider audience about public involvement and research, using a broad range of approaches that are accessible and appealing.

Nine of the reviews discussed communication as being important to ensure PPI activity is successful [7, 28–29, 31, 36, 38–39, 42]. Various attributes of good communication were discussed with the main points listed in Table 7 below, and explicitly addresses the problem area of Communications.

**Table 7:** Solutions - Communications

Attribute	Study/Studies	Examples of reasoning
Listen, act and feed back	28, 31, 38–39	Helps address issues such as power (40), let people know what you are doing with their suggestions and why (28), ensures accountability (31)
Ongoing/ regular updates	29, 36, 41	Contribute to motivation and engagement, and to foster satisfying partnerships (36)
Creating space to voice concern/ open communication climate	28, 36	Contribute to motivation and engagement, and to foster satisfying partnerships (36)
Avoid/ translate jargon	28–29, 36	Ensuring everyone understood and felt comfortable and confident to engage in meaningful dialogue (36)
Use different materials (not just written reports etc.)	36, 38, 41	Ensure people with different levels of literacy can participate (36)
Sharing information, experiences and knowledge	7, 38	Across all groups involved (7)
Clarifying and agree expectations upfront	28, 36	Could avoid conflicts, demotivation, dissolution of partnerships, or frustration in situations where stakeholders could perceive a lack of concrete actions (36), patients are ‘partners’ not ‘are involved’ (28)
Have stakeholders lead groups	36	But be careful they include all groups in the discussion (36)

### Impact

Solution: Seek improvement by identifying and sharing the difference that public involvement makes to research. Understand the changes, benefits and learning gained from the insights and experiences of patients, carers and the public.

Seven of the reviews discussed impact [7, 24, 28, 36, 38–39, 42–43]. The general theme was that impact needs to be better evaluated throughout the whole research lifecycle. It was noted that this is an area where the existing literature is scant and current working practices are perceived to be lacking in terms of rigour. Most studies focused on the impact of PPI activity on participants, researchers or the research itself – rather than setting out to formally assess what works to make PPI activity successful. Moreover, there is much still to be decided about what impact may be reasonably expected to be seen. Brett et al. [44] noted particularly the lack of any evidence of any financial analysis and Jones et al. [45] suggested that the use of contemporaneous real time data concerning PPI within surgical trials, currently lacking, could be made use of. Furthermore, it is not always possible to predict the impact of the involvement, as we are not always able to determine or anticipate potential problems or issues raised by PPI as the study progresses. One important contextual factor consistent throughout the research development is the researcher themselves, their previous experiences, skills, knowledge and beliefs. The researcher experiences the impact of PPI as the research develops [46].

Evaluating impact through continuous assessment and feedback was seen to be important in order to ensure ongoing involvement, to identify best practice and areas for improvement, and to make sure that the experience is working for everyone involved. In addition to evaluating the process of PPI within health research, it was also noted that the impact of findings that are translated to real world settings, and ideally the contribution of PPI activity to that impact, should also be evaluated.

It is important to note that impact can be positive or negative and that impact may happen in a complex way and to a range of areas, for example, impact on the research, on the research outcomes, on the researchers, on the PPI members, on the wider community and stakeholders.

### Other issues

Interestingly considering the topic of the reviews, the use of PPI members in the reviews was not universal.

- 9 reviews described PPI throughout the review process;
- 3 reviews took their findings to PPI members for discussion;
- 3 reviews made use of external panels or organisations;
- Single reviews reported utilising PPI at specific stages:
  - To identify research questions;
  - Reviewing protocol;
  - During execution and translation;
  - Reviewing the process;
  - Feedback from stakeholder but stage not stated;
- 2 reviews mentioned that there had not been any PPI in the review;
- 9 reviews did not mention PPI in their own review process at all.

Few of the reviews detailed the studies discussed within them in terms of types of PPI or in terms of stages of research although most included some discussion of these areas in general terms. Dawson et al. [47] is one exception where the studies are clearly detailed in terms of what PPI groups or individuals were involved in various tasks.

There was no consistent terminology used for either types of PPI or stages of research. There has been some attempt to categorise these at a national level. For example, in the UK, INVOLVE distinguished between three PPI approaches: consultation, collaboration and user-led; while Health Canada divides PPI into five stages: inform or educate, gather information, discuss, engage and partner (Pii)[22].

Crocker et al. [48] describes the types of involvement covered in the studies to range ‘from one person to many people or whole patient organisations, from one-off involvement in a particular aspect of the trial (for example, reviewing draft information for patients or recruiting participants from their communities) to involvement throughout the trial (for example, as members of a trial steering committee), and from involvement with no decision making power (for example, as advisers) to involvement in decision making as equal partners’. Some examples of the stages of research where PPI was included are summarised in Table 8.

**Table 8:** Examples of stages of research where PPI was included

Wilsher (27)	Domecq (30)	Pii (22)
• Identify/prioritise • Design • Grant development • Undertake/ Manage • Analysing/ interpret • Dissemination • Monitoring/ evaluation	1) Preparatory phase (agenda setting, prioritization of research topics and funding). 2) Execution phase (study design & procedures, study recruitment, data collection, and data analysis). 3) Translation phase (dissemination, implementation, and evaluation).	1. Development of research focus Research definition Research prioritization 2. Development of research design Method development Study design development 3. Recruitment Recruitment strategy Recruitment 4. Data generation 5. Data processing/ Analysis 6. Research dissemination Dissemination Dissemination strategy

## Discussion

This review of reviews set out to see what evidence there was concerning optimising patient and public involvement specific to population health research. The novelty in this review of reviews is twofold: firstly, that the findings have been framed by the UK Standards and secondly, that the challenges have been matched against potential solutions. The UK Standards were used to map evidence of successful development, implementation and evaluation of patient and public involvement and then translated into tools containing best practice guidance to further drive-up standards in the conduct of PPI in population health research (see supplementary file 4 for new guidance and tools for use in population health research).

Most reviews were about PPI activity in specific thematic healthcare areas or in general health and social care research but the details of the studies included in the reviews makes it clear that many studies included were of direct relevance to population health research. The findings are, therefore, both generic across health and social care research as well as providing useful evidence-based suggestions as to what works in PPI in population health research.

### Comparing findings with recently published primary studies

Looking at recently published primary studies we found several of interest, mainly around data-driven population health research. The principles that emerge from these studies fit well with the findings of the review of reviews, but also suggest that there are a variety of approaches through which PPI can be addressed and improved. We summarise recent primary studies in Table 9.

**Table 9:** Recent population health primary studies addressing PPI.

Population Health Specific PPI Challenge Area	Study	Aspects of note
Data-driven	Johnson et al. [49]	• There is little guidance on how to meaningfully involve the public in big data research. • Involvement in big data research is significantly limited in comparison with other study designs. • May be because common approaches to public involvement adopted in primary data research are not appropriate within big data analysis studies. • The highly data driven discussions that underline this type of research can present a barrier to public involvement. • There is now growing recognition that public involvement in big data research requires special considerations.
Data-driven	Hobbs et al. [50]	Enhance public forum members’ personal development in data-intensive health research through a personal development portfolio: • Personal Profile - Personal details including education, qualifications and employment • Relevant Experience - Volunteering and personal experience • Training Record - Training events attended and events where been trainer or facilitator • Personal statement - Overall description of skills and experience they may have gained from involvement activities • Involvement activities - Summary of each activity, skills and experience gained, evidence such as certificates or feedback and personal reflections on their involvement in this activity • References - Details of relevant individuals and how known to the public contributor.
Data-driven	‘Consensus Statement on Public Involvement and Engagement with Data Intensive Health Research’ [51]	Key Principles for Public Involvement and Engagement in Data-Intensive Health Research – 1. Have institutional buy-in 2. Have clarity of purpose 3. Be transparent 4. Have two-way communication 5. Be inclusive and accessible to broad publics 6. Be ongoing 7. Be designed to produce impact 8. Be evaluated.
Complexity	Van Voorn et al. [52]	• Involving patients in health economic research will require a serious investment of time and money for patients to get to a level at which they can contribute. • Patients need to be able to ‘rise above’ their condition - to find an interest in the material itself and have an objective view. • Proper selection procedures will have to be developed.
Representation & data-driven	Jewell et al. [53]	Report on the setting up of a service user and carer advisory group supporting data linkage in mental health research. • The general public feel that the complexities of data linkage research may be difficult to explain in lay terms and that patients and the public have limited knowledge about data, anonymisation, aggregation, and the regulations surrounding these. • Training sessions were set up for all new group members. Training sought to provide members with information about data linkage, including the information governance procedures in place to protect the personal data of service users.

The specific aspect of longer-term duration that is often typical of population health studies is best illustrated through the examination of existing longitudinal studies as case studies. Longitudinal studies involve repeated observations of the same subjects, allowing researchers to analyse change at the individual level. Such studies typically last decades, such as the 1970 British Cohort Study [54] or the Medical Research Council National Survey of Health and Development [55] which started in 1946.

Considering involvement in longitudinal studies, one approach is that used by the ALSPAC study could be considered an exemplar of best practice [56]. Based at the University of Bristol, the Avon Longitudinal Study of Parents and Children (ALSPAC), also known as Children of the 90s, is a world-leading birth cohort study. One of the governance aspects of the study is the original cohort advisory panel (OCAP) which is made up of more than 30 study participants who meet bi-monthly to provide insights and advice on study design, methodology and acceptability for participants. The group has been running since 2006.

The main aims of the OCAP group are:

- To represent the cohort of original study children;
- To review study documentation and provide feedback to CO90s staff;
- To represent and convey participants’ opinions about planned research exercises.

Taken collectively, these supplementary sources suggest that certain solutions identified in the reviews, such as good communication and tailored training, are even more vital to PPI in population health research. One thing that emerges strongly from these studies is the idea that PPI selection and recruitment for population health research projects needs to be very carefully considered.

### Fit of the UK Standards

The UK Standards proved to be a coherent framework for capturing solutions and no solution was offered that did not fit in to one of the six categories. It was, however, notable that two standards were less discussed than others: Governance and Impact. Capturing, measuring and illustrating the impact of PPI within the entire lifespan of a project is an issue that has not yet been resolved but is currently being addressed by various organisations. The absence of Governance may be a result of language use, as some attributes of Working Together were relevant in terms of this standard but were not couched in terms of Governance specifically. It was also interesting to see that Communications is a UK Standard separate from Working Together, as it was something that could be seen to be an integral part of Working Together. One further point of consideration is that it could be considered that the aspirational end point of PPI would be that any involvement would become so integral to the project that it would be difficult to unpick whose contribution had led to an impact or outcome not originally anticipated.

In addition, peer reviewer feedback on this manuscript highlighted the notion of ‘representation’ or ‘representativeness’ as a very contentious subject in the context of public involvement in population health research. The UK standards refer to offering opportunities to people and groups depending on research needs but does not mention engaging with whole communities as would be expected in a population health research context. There was a strong view expressed by one peer reviewer that ‘*no one else is expected to be representative of a community in a research team so why should we expect this of our public contributors? I actually think public/population health research provides an excellent opportunity to move away from this by placing a greater emphasis on working with and co-producing with communities as opposed to individuals.’* We agree with this view and support the type of PPI engagement advocated by the peer reviewer for population health research.

### Strengths and limitations of the review of reviews

The review of reviews was carried out using systematic processes and following production of an a priori protocol. Not all data were however complete for all reviews and there was a wide variety within the reviews that did report data. For example,

- The number of studies reported in each review varied from 4 [41] to 251 [39];
- Years searched ranged from time periods defined by the previous decade [22] to those that searched back to the inception of the databases searched [30];
- Geography also varied but, of those reviews which gave details of geographical settings, the vast majority of the studies were from the UK (n = 292), followed by the USA (n = 95) and then other areas: Canada (n = 38), Europe (n = 29), Australia (n = 25), and other countries or multiple site studies (n = 17).

The reviews covered a range of diagnostic areas ranging from generic health and social care [18] or clinical trials [47] to condition specific areas such as diabetes [37] or palliative care [21]. Although a broad range of conditions were covered, this review did not focus on condition-specific aspects which could act as challenges for involvement. However, this was not within the remit of this review which had a greater focus on PPI in population health research. Interestingly there were few reviews based on demographic groups who are generally acknowledged to be under-represented in healthcare decision making:

- There was one review for ethnic minority communities [19] and the geography of the studies included were mainly in the United States.
- There was one review for Older People [24] which covered nine qualitative articles. Arguably studies around dementia and palliative care may be relevant to this demographic but that cannot be assumed.
- There were three reviews for Children and Young People – all of which had a specific focus rather than looking at the involvement of Children and Young People in PPI more generally:
  - Children and Families in Pediatric Health Research [23];
  - Disabled children [25];
  - Paediatric Intensive Care [41].

On the positive side, Malterud et al. [57] however noted the usefulness of ‘two articles which describe in detail how individuals with limited literacy abilities can be supported to analyse and communicate such processes’.

## Conclusions

There are several important areas of PPI activity that require further research. With regards to Population Health research, there remain gaps in knowledge about how to ensure key specific attributes of this type of research are addressed for PPI team members – particularly around how to deal with complexity and the data-driven nature of the research. Looking at the UK Standards when mapped against the findings, it is clear that there is a lack of current research or clarity around Governance and Impact. There could also be more research done about PPI with under-represented groups. The new tools containing best practice guidance produced from the synthesis and examples of resources are designed to help population health researchers to facilitate better PPI and in turn to conduct better research.

## Electronic supplementary material

Below is the link to the electronic supplementary material.

Supplementary Material 1

Supplementary Material 2

Supplementary Material 3

Supplementary Material 4

## Data Availability

All data generated or analysed during this study are included in this published article [and its supplementary information files].

## Abbreviations

ALSPAC: Avon Longitudinal Study of Parents and Children
CO90s: Children of the 90s
CPH: National Centre for Population Health and Wellbeing Research
HCRW: Health and Care Research Wales
HE: Health Economics
HTA: Health Technology Assessment
IKT: Integrated knowledge translation
OCAP: Original Cohort Advisory Panel
PPEET: Public and Patient Engagement Evaluation Tool
PPE: Patient and Public Engagement/ Public and Patient Engagement
PPI: Patient and Public Involvement / Public and Patient Involvement

## Footnotes

1 When a project outgrows its original remit without any additional resources being available.
