# Supplementary material for: A review of reviews exploring patient and public involvement in population health research and development of tools containing best practice guidance
Source: BMC Public Health. 2023 Jun 30;23:1271. doi: 10.1186/s12889-023-15937-9 (PMC10311710; doi:10.1186/s12889-023-15937-9)
Supplement: Supplementary file 2 — Supplementary Material 2 [file 12889_2023_15937_MOESM2_ESM.docx]

# **Quality Assessment**

|  | **Did the review address a clearly focused question?** | **Did the authors look for the right type of papers?** | **Do you think all the important, relevant studies were included?** | **Did the review’s authors do enough to assess quality of the included studies?** | **If the results of the review have been combined, was it reasonable to do so?** | **How precise are the results?** | **Can the results be applied to the local population?** | **Were all important outcomes considered?** | **Are the benefits worth the harms and costs?** |
| --- | --- | --- | --- | --- | --- | --- | --- | --- | --- |
| **Studies** | **Agreed** | **Agreed** | **Agreed** | **Agreed** | **Agreed** | **Agreed** | **Agreed** | **Agreed** | **Agreed** |
| Bailey (25) | Y | Y | Y | Y | Y | CT | Y | Y | Y |
| Baines (38) | Y | Y | Y | Y | Y | % | Y | Y | Y |
| Baldwin (24) | Y | Y | Y | Y | Y | CT | Y | Y | Y |
| Bethell (29) | Y | Y | N | N | Y | CT | Y | Y | Y |
| Boote (31) | Y | Y | N | N | Y | CT | Y | Y | Y |
| Brett (18) | Y | Y | Y | Y | Y | CT | Y | Y | Y |
| Brett (26) | Y | Y | Y | Y | Y | CT | Y | Y | Y |
| Brett (44) | Y | Y | Y | Y | Y | CT | Y | Y | Y |
| Camden (36) | Y | Y | Y | N | Y | CT | Y | Y | Y |
| Chambers (40) | Y | Y | Y | Y | Y | CT | Y | Y | Y |
| Crocker (48) | Y | Y | N | N | Y | % | Y | Y | Y |
| Dawson (19) | Y | Y | N | Y | Y | CT | Y | Y | Y |
| Domecq (30) | Y | Y | Y | Y | Y | CT | Y | Y | Y |
| Fergusson (34) | Y | Y | N | N | Y | % | Y | Y | Y |
| Flynn (23) | Y | Y | N | N | Y | CT | Y | Y | Y |
| Harris (37) | Y | Y | Y | N | Y | CT | Y | Y | Y |
| Jagosh (35) | Y | Y | N | N | Y | CT | Y | y | Y |
| Jones (45) | Y | Y | Y | Y | Y | CT | Y | Y | Y |
| Malterud (57) | Y | Y | N | Y | Y | CT | Y | Y | Y |
| Manafo (7) | Y | Y | N | Y | Y | CT | Y | Y | Y |
| Menzies (41) | Y | Y | N | Y | Y | CT | Y | Y | Y |
| Miah (43) | Y | Y | Y | N | Y | CT | Y | Y | Y |
| Nunn (32) | Y | Y | N | N | Y | % | Y | Y | Y |
| Pii (22) | Y | Y | CT | Y | Y | CT | Y | Y | Y |
| Price (28) | Y | Y | Y | Y | Y | CT | Y | Y | Y |
| Sangill (33) | Y | Y | Y | Y | Y | CT | Y | Y | Y |
| Scholz (21) | Y | Y | Y | Y | Y | CT | Y | Y | Y |
| Shippee (42) | Y | Y | Y | N | Y | CT | Y | Y | Y |
| Vaughn (39) | Y | Y | N | N | Y | CT | Y | Y | Y |
| Wilsher (27) | Y | Y | CT | N | Y | CT | Y | Y | Y |
| Zych (20) | Y | Y | N | Y | Y | CT | Y | Y | Y |

**Key:**

Y = Yes

N = No

CT = Can’t tell

% = precision given a percentage score
