# Supplementary material for: A review of reviews exploring patient and public involvement in population health research and development of tools containing best practice guidance
Source: BMC Public Health. 2023 Jun 30;23:1271. doi: 10.1186/s12889-023-15937-9 (PMC10311710; doi:10.1186/s12889-023-15937-9)
Supplement: Supplementary file 4 — Supplementary Material 4 [file 12889_2023_15937_MOESM4_ESM.docx]

**New guidance and tools for use in population health research**

**Tool 1. Best practice guidance to drive up quality in PPI mapped against the specific challenges of conducting population health research**

| **Representation:**   - Use a variety of methods and partners to recruit a range of PPI members with different knowledge, skills and experience at relevant points in the project lifecycle. - To fit better with wider community context, include relevant stakeholders and agencies also clinicians, charities, specialist support services plus patient and advocacy groups, be proactive and go out and get involved, don't expect people to come to you, build more meaningful relationships with the target population.   **Complexity & Data-Driven:**   - Allow for the formal development of knowledge and skills, supporting PPI team members to be informed and make informed decisions and to understand specific parts of the research process and/or context. - Make learning relevant to the specific context of the research and at the appropriate level for the PPI team member to allow full participation and to build participant capacity. - Provide training in research components to give PPI team members confidence in their involvement and to explain ‘rules’ and constraints of research. - Use a variety of methods such as supervision, mentoring, formal training, workshops and team-based sessions, include everyone on the team if possible.   **Duration:**  All of the aspects above are important and need to be considered dynamically throughout the duration of the project.  Generally, researchers should plan for an evolving and varied PPI contribution over time with different PPIs being brought into the project to meet the specific requirements at the time. It may however be useful to maintain a continuous thread of a core group of PPI team members to ensure continuity.  Additional key components regarding the typically longer duration of population health projects are:   - Communication – clear, accessible and timely communications using different methods as appropriate - Governance – clarity of roles and structures to support the project throughout its life - Relationship building – take time to build strong, meaningful relationships to improve PPI retention and understanding - Resources – ensure sufficient time, financial and practical resources are available for the duration of the project including lead in and post-project activity - Staff continuity – mitigating for changes in personnel, whether principal investigator, key researchers or PPI members - Flexibility – recognising that projects and external drivers do need to adapt to changes in a measured and managed way - Continuous evaluation and feedback – particularly important in longer projects to ensure that everything stays on track and to ensure that any issues are picked up sufficiently early to allow change. |
| --- |

## **Tool 2. Best practice guidance for driving up the quality of PPI in population health research mapped against the UK Standards for PPI.**

| **Inclusive opportunities** | |
| --- | --- |
| **Heading** | **Aspects to consider** |
| Accessibility | Venues should be located for the ease of the participants, accessible and meetings should be timed appropriately and include communication aids, breaks and refreshments as appropriate for individual and collective needs. |
| Methods of engagement | Online technology could assist people to be included who are often excluded from traditional engagement e.g. those with illness, time poor, caring responsibilities. Especially when working with disabled children and young people, be flexible for different abilities and ages and offer choice, use variety of methods. |
| Incentives | Provide incentives for participants. |
| Anonymity | Ensure anonymity for participants if required or requested. |
| Representation and/or diversity | Use variety of methods and partners to recruit a range of participants, understand different motivations and gain insight into the community, view differing perspectives as valuable, recognise and address issues concerning diversity, avoid tokenism. |
| Community consultation | To fit better with wider community context, include relevant stakeholders and agencies also clinicians, charities, specialist support services plus patient and advocacy groups. Be proactive and go out and get involved, don't expect people to come to you, build more meaningful relationships with target population. |
| Safe environment | Consider whether a trusted adult or facilitator is useful. |
| Recruit well | Fit skills and experiences to the project, recruit through a variety of ways, need to be not just representative but also be able to be collaborative. |
| **Working together** | |
| **Heading** | **Aspects to consider** |
| Resources | Consider different aspects of resourcing such as budget/ funding, building in sufficient time to build relationships, communicate etc., consider using existing PPI resources or groups where available, plan into proposals, tailor to project. |
| Clarity | Ensure clarity of various aspects including: roles, expectations, structures. |
| Workload | Prevent workload getting too much. |
| Preparation | Prepare in advance as much as possible. |
| Staff continuity | Mitigate and prepare for staff turnover but avoid if possible. |
| Scope creep | Prevent or manage scope creep. |
| Relationships | Manage conflict, take time to build partnerships built on joint ownership, trust, respect and transparency, empower PPI members by sharing power and knowledge, explore risks together, consider capacity of PPI members. |
| Engagement | Conduct engagement early on, provide multiple and varied opportunities as appropriate. Always acknowledge contributions. |
| Flexibility | Acknowledge that confidence, personal circumstances and capacity may change over time, keep tasks flexible and include time for training and questions. Be flexible generally in attitude and approaches to the project. |
| Ethical concerns | Consider and address possible ethical concerns throughout and understand that they may not always be obvious. |
| Challenging the establishment | Have honest conversations about motivations of team members, including PPI and other stakeholders, on all sides. |
| **Support and learning** | |
| **Heading** | **Aspects to consider** |
| Practical support | Think about details e.g. childcare, food, location, transport, compensation, timings, and have strategies for when people are ill/ can’t take part. |
| Structural support | Make sure key project individuals support PPI, provide structures that support PPI, include relevant institutions such as charities, volunteer groups etc. |
| Formal knowledge | Encourage formal development of knowledge and skills, supporting participants to be informed and make informed decisions and to understand specific parts of the research process and/or context. |
| Learning as appropriate | Make learning relevant to the specific context of the research and at the appropriate level for the PPI member to allow full participation and to build participant capacity. |
| Research methods | Ensure access to training in research components to give confidence in their involvement and to explain ‘rules’ and constraints of research. |
| Emotional support | Recognise that experiences may be upsetting, provide safe spaces, provide consistent feedback and support, consider how to deal with anxiety. |
| Share knowledge | Acknowledge that knowledge and experience flow both ways and make ways to facilitate that flow. |
| Specific support | Ensure support specific to topic area and to their individual involvement. |
| Variety | Use a variety of methods such as supervision, mentoring, formal, workshops and team based, include everyone on the team if possible. |
| **Governance** | |
| **Heading** | **Aspects to consider** |
| Shared decision making | Create shared decision making (at every level), power and leadership. |
| **Communications** | |
| **Heading** | **Aspects to consider** |
| Have stakeholders lead groups | But be careful they include all groups in the discussion. |
| Ongoing/ regular updates | Provide regular update to contribute to motivation and engagement, and to foster satisfying partnerships. |
| Be open | Create space to voice concern/ open communication climate. |
| Avoid/ translate jargon | Ensure everyone understands and feels comfortable and confident to engage in meaningful dialogue. |
| Use different materials (not just written reports etc) | Ensure people with different levels of literacy can participate. |
| Listen, act and feedback | Good communication channels help address issues such as power, let people know what you are doing with their suggestions and why, ensure accountability. |
| Sharing information, experiences and knowledge | Sharing should be common across all groups involved. |
| Clarifying and agreeing expectations upfront | Clarify expectations to avoid conflicts, demotivation, dissolution of partnerships, or frustration in situations where stakeholders could perceive a lack of concrete actions, patients are 'partners' not 'are involved'. |
| Prioritising personal experience | Ensure people are clear on their role. |
| Scepticism | Avoid creating an atmosphere of doubt. |
| **Impact** | |
| **Heading** | **Aspects to consider** |
| Better evaluation | Develop and use evaluation throughout the project which focuses on PPI activity. |
| Continuous assessment and feedback | Impact should be measured across all groups involved. |
| Impact of project, PPI role and translation to real world situations | Monitor and reflect on impact throughout project. |

## What is missing?

Not all challenges in conducting PPI in population health Research were matched by solutions as shown in Tool 3. Challenges not matched to solutions are in bold and have been allocated to an appropriate UK Standard. Tool 3 is designed to whelp researchers identify where additional research and solutions are required.

Tool 3. **Challenges of facilitating good PPI in health research not matched against a solution are shown in bold.**

| **Challenges** | | **Solutions** | |
| --- | --- | --- | --- |
| **Heading** | **Sub-heading** | **Attribute** | **UK Standard** |
| Resources | Lack of budget | Resources | Working Together |
|  | Lack of time | Resources | Working Together |
|  | Emotional burden | Emotional support | Support and Learning |
|  | Complicated logistics/ infrastructure | Structural support | Support and Learning |
|  | **Workload too high** | - | **Working Together** |
|  | **Lack of incentives** | - | **Inclusive Opportunities** |
|  | **Lack of preparation** | - | **Working Together** |
|  | **Lack of staff continuity** | - | **Working Together** |
|  | Lack of support for PPI members | Various | Support and Learning |
|  | **Scope creep** | - | **Working Together** |
| Conflict and control | Allowing power to be shared with PPI | Relationships | Working Together |
|  | Expectations (from all sides) | Clarity | Working Together |
|  | Conflicting perspectives | Relationships | Working Together |
|  | A culture of researchers vs PPI | Relationships | Working Together |
|  | **Ethical concerns** | - | **Working Together** |
|  | **Challenging the establishment** | - | **Working Together** |
|  | Differences within communities | Community consultation | Inclusive Opportunities |
|  | Accepting the legitimacy of PPI | Clarity | Working Together |
|  | **Prioritising personal experience** | - | **Communications** |
|  | **Scepticism (from all sides)** | - | **Communications** |
|  | Unresolved conflict | Relationships | Working Together |
| Knowledge | Processes | Structural support | Support and Learning |
|  | Language/ jargon | Avoid jargon | Communications |
|  | Lack of skills or training | As appropriate | Support and Learning |
|  | Administration issues | Structural support | Support and Learning |
|  | Working practices | Structural support | Support and Learning |
| Representation | Reflecting the diversity of affected populations | Representation and/ or diversity | Inclusive Opportunities |
|  | Tokenism of PPI (aka box-ticking) | Representation and/ or diversity | Inclusive Opportunities |
|  | Getting early-stage involvement | Engagement | Working Together |
|  | Involving children | Methods of engagement | Inclusive Opportunities |
|  | **Protecting anonymity** | - | **Inclusive Opportunities** |
|  | Accessibility (venues) | Accessibility | Inclusive Opportunities |
| Communication | Lack of meaningful and timely communication leading to disenfranchisement | Ongoing/ regular updates | Communications |
|  | Difficulty reporting impact of PPI | Evaluation | Impact |
|  | Building relationships to sustain involvement | Relationships | Working Together |
|  | Transparency of research process | Relationships | Working Together |
|  | Building trust (on all sides) | Relationships | Working Together |
|  | Different values within the team | Relationships | Working Together |

Tool 4. Examples of resources, methods and tools for integrating and evaluating PPI. Primarily UK focussed – but many guidance documents and tools will have international relevance.

| Tool | Description |
| --- | --- |
| UK Standards for Patient and Public Involvement (1) | Contains 6 standards against which to conduct and evaluate PPI. |
| [FOR Equity](https://eur01.safelinks.protection.outlook.com/?url=https%3A%2F%2Fhealthandcareresearchwales.us6.list-manage.com%2Ftrack%2Fclick%3Fu%3Dc607c1a601d761a09d4312aa1%26id%3D90a0b3e13b%26e%3D330f58d51d&data=05%7C01%7Cjane.noyes%40bangor.ac.uk%7Cdb31fb24c9964058deae08dab8d39161%7Cc6474c55a9234d2a9bd4ece37148dbb2%7C0%7C0%7C638025516093385071%7CUnknown%7CTWFpbGZsb3d8eyJWIjoiMC4wLjAwMDAiLCJQIjoiV2luMzIiLCJBTiI6Ik1haWwiLCJXVCI6Mn0%3D%7C3000%7C%7C%7C&sdata=8kXN3sABS7erxeLRXlxuyL9if1F8xChf%2BwdfYeX29Vg%3D&reserved=0) (2) | Contains tools and resources to help researchers integrate an equity lens into their research, and consider how people with lived experience and policy or practice expertise can help in this process. It contains:   - a guidance inventory - Health Inequalities Assessment Toolkit (HIAT) - an equity resources library |
| [INVOLVE \| people at the heart of decision-making (3)](https://involve.org.uk/) | UK's public participation charity, on a mission to put people at the heart of decision-making. Maintains a library of resources, methods, case studies and a blog |
| [Involving the public in your research \| Health Care Research Wales](https://healthandcareresearchwales.org/researchers-support-and-guidance-researchers-develop-research-idea/involving-public-your-research) (4) | - Read more on [how to engage with members of the public about getting involved with your research](https://healthandcareresearchwales.org/sites/default/files/2020-10/public_inv_key_messagesv2.0_Feb20_en.pdf) - Find out about the [three categories of public involvement](https://healthandcareresearchwales.org/sites/default/files/2020-10/Three_categories_of_involvement_A4_eng_0.pdf) and which one is suitable for your research - Public involvement training: We have developed an [online introduction to public involvement](https://healthandcareresearchwales.org/training/introduction-public-involvement-research) short training course which can be accessed by researchers and members of the public involvement community. An NIHR library of useful public involvement courses can be found here <https://learningforinvolvement.org.uk/> which will eventually include those developed in and for Wales   **Appointing members of the public**  Once you have appointed members of the public to get involved in your research, the following tools and templates will be useful:   - [Appointment checklist for researchers](https://healthandcareresearchwales.org/sites/default/files/2023-04/Appointment_checklist_v4.0_30.03.2022.docx) - a checklist to assist in progressing with the next steps for meaningful public involvement. - [Code of conduct template](https://healthandcareresearchwales.org/sites/default/files/2023-04/Code_of_conduct_v1.0_27.03.2019.docx) - this template can be adapted and populated to meet the needs of your organisation. - [Terms of reference template](https://healthandcareresearchwales.org/sites/default/files/2023-04/Terms_of_reference_templatev1.0_April_2022.docx) - This template is designed to help you develop terms of reference for a group such as a public involvement advisory groups or research panels - [Problem solving procedure](https://healthandcareresearchwales.org/sites/default/files/2023-04/Problem%20Solving%20Procedure%20v2.0.pdf) - involvement of the public is not employment and usually it is a positive experience for all concerned. Sometimes things go wrong, this procedure should help and is based on advice from the Wales Centre for Voluntary Action (WCVA). - [Payment for Public Involvement in Health and Care Research: A guide for organisations on determining the most appropriate payment approach](https://healthandcareresearchwales.org/sites/default/files/2022-06/Payment_for_Public_Involvement_in_Health_and_Care_Research_eng.pdf) - [Benefits Advice Service for involvement](https://healthandcareresearchwales.org/sites/default/files/2022-06/Benefits_advice_service-June_2022_eng.pdf) - a service that can provide advice about the impact of receiving payment for time on members of the public who are in receipt of benefits   **Training and support**  We provide training and support to make sure you can get the most out of involving the public in your research.   We have developed an [online introduction to public involvement](https://healthandcareresearchwales.org/training/introduction-public-involvement-research) short training course which can be accessed by researchers and members of the public involvement community. An [NIHR library of useful public involvement courses](https://learningforinvolvement.org.uk/) will eventually include those developed in and for Wales |
| [Public and Patient Engagement Evaluation Tool (PPEET),](https://ppe.mcmaster.ca/resources/public-and-patient-engagement-evaluation-tool/) developed by McMaster University in Canada (5) | Provides three questionnaires that examine involvement from the point of view of the participant, the project or the organisation |
| General guidance provided on the on the National Institute for Health Research (NIHR) [PPI (Patient and Public Involvement) resources for applicants to NIHR research programmes \| NIHR](https://www.nihr.ac.uk/documents/ppi-patient-and-public-involvement-resources-for-applicants-to-nihr-research-programmes/23437) (6) | Guidance for researchers on PPI [NIHR has developed guidance for researchers on how patients and the public can be involved.](https://www.nihr.ac.uk/documents/briefing-notes-for-researchers-public-involvement-in-nhs-health-and-social-care-research/27371) Payment and recognition for PPI [NIHR also provide guidance on reward and payment for involvement.](https://www.nihr.ac.uk/documents/payment-guidance-for-researchers-and-professionals/27392) Co-production [Also you can find NIHR guidance on co-producing a research project.](https://www.learningforinvolvement.org.uk/?opportunity=nihr-guidance-on-co-producing-a-research-project) Providing feedback to public contributors [Useful, practical and evidence-based guidance (.PDF)](https://www.clahrc-eoe.nihr.ac.uk/wp-content/uploads/2016/05/Guidance-for-Researchers-PPI-Feedback_2018.pdf) is available on providing effective two-way feedback between researchers and public contributors. Online Public Reviewing Course You may find the [online interactive course for patient/public reviewers](https://www.learningforinvolvement.org.uk/an-interactive-course-for-new-and-experienced-patient-public-reviewers-of-health-and-social-care-research/) useful when considering how to design the PPI in your project. It will describe how patient and public reviewers and public committee members assess research plans. |
| Evaluation guidance provided on the National Institute for Health Research (NIHR) [PPI (Patient and Public Involvement) resources for applicants to NIHR research programmes \| NIHR](https://www.nihr.ac.uk/documents/ppi-patient-and-public-involvement-resources-for-applicants-to-nihr-research-programmes/23437) (6) | Suggests the following categories of evaluation:   - Impact log – a simple method of recording outcomes - ‘Cube’ framework – used to evaluate the process or quality of involvement - Public Involvement Impact Assessment Framework (PiiAF) – more comprehensive method consisting of two parts 1) planning involvement in a research project, 2) designing a plan to evaluate the impact of involvement - Realist evaluation – identifies what works for whom in what circumstances to achieve which outcomes, and how and could be applied to evaluate the impact of PPI in research studies |
| [The Public Involvement in Research Impact Toolkit](https://www.cardiff.ac.uk/marie-curie-research-centre/patient-and-public-involvement/public-involvement-in-research-impact-toolkit-pirit) (PIRIT) (7) | Includes tools to track and report the impact of PPI in research studies. |
| Guidance for Reporting Involvement of Patients and the Public (GRIPP2) checklist (8) | Assesses reporting of PPI |
| Guidance Document: Evaluating public involvement in research (9) | Presents and discusses approaches to evaluating PPI |

# **References**

1. The UK Standards: Setting the scene. UK Standards for Public Involvement. [cited 2023 April 14]. Available from: <https://sites.google.com/nihr.ac.uk/pi-standards/standards/setting-the-scene>
2. For Equity. [cited 2023 April 14]. Available from: https://forequity.uk/
3. INVOLVE. People at the heart of decision making. [cited 2023 April 14]. Available from: <https://involve.org.uk/>
4. Involving the public in your research. Health and care research Wales. [cited 2023 April 14]. Available from: <https://healthandcareresearchwales.org/>
5. Abelson J, Li K, Wilson G, Shields K, Schneider C, Boesveld S. Supporting quality public and patient engagement in health system organizations: development and usability testing of the Public and Patient Engagement Evaluation Tool. Health Expectations. 2016;19(4):817-827. <https://doi.org/10.1111/hex.12378>
6. [PPI (Patient and Public Involvement) resources for applicants to NIHR research programmes. National Institute for Health and Care R](https://www.nihr.ac.uk/documents/ppi-patient-and-public-involvement-resources-for-applicants-to-nihr-research-programmes/23437)esearch. [cited 2023 April 14]. Available from: <https://www.nihr.ac.uk/documents/ppi-patient-and-public-involvement-resources-for-applicants-to-nihr-research-programmes/23437>
7. Public Involvement in Research Impact Toolkit (PIRIT). Marie Curie Research Centre. [cited 2023 April 14]. Available from: https://www.cardiff.ac.uk/marie-curie-research-centre/patient-and-public-involvement/public-involvement-in-research-impact-toolkit-pirit
8. Staniszewska S, Brett J, Simera I, Seers K, Mockford C, Goodlad S et al. GRIPP2 reporting checklists: tools to improve reporting of patient and public involvement in research BMJ 2017; 358 :j3453 doi:10.1136/bmj.j3453
9. Kok M (2018) Guidance Document: Evaluating public involvement in research. UWE Bristol. Available from: http://www.phwe.org.uk/wp-content/uploads/Guidance-on-evaluating-Public-Involvement-in-research.pdf
